# Carbapenem-Resistant *Klebsiella pneumoniae* Isolated From a Patient in a Midwestern U.S. Hospital With a History of Indian Travel: Therapeutic Strategies and Clinical Outcomes

**DOI:** 10.1155/crdi/8155592

**Published:** 2025-01-31

**Authors:** Christopher Phan, Kristen Tsai, Christian M. Gill, Robin Chamberland, Christian Hendrix, Rong Hou

**Affiliations:** ^1^Department of Internal Medicine, Saint Louis University School of Medicine, St. Louis, Missouri 63110, USA; ^2^SSM Health Saint Louis University Hospital, St. Louis, Missouri 63110, USA; ^3^Department of Pathology, Saint Louis University, St. Louis, Missouri 63110, USA; ^4^Division of Infectious Diseases, Allergy and Immunology, Department of Internal Medicine, Saint Louis University School of Medicine, St. Louis, Missouri 63104, USA

**Keywords:** antimicrobial resistance, bacteremia, carbapenemase, carbapenem-resistant *Klebsiella pneumoniae*, international travel

## Abstract

Carbapenemases have had increasing prevalence within the United States and worldwide. Here, we present a case of carbapenem-resistant *Klebsiella pneumoniae* (CRKP) which is unique due to the rarity of multiple mechanisms of resistance within the *Klebsiella pneumoniae* harboring New Delhi metallo-*β*-lactamases (NDM), oxacillinase-48 (OXA-48)-like, and cefotaximase (CTX-M) resistance genes, detected in a patient following an international travel. This case demonstrates the need for a multidisciplinary approach to optimize the treatment of multidrug-resistant Gram-negative organisms.

## 1. Introduction

The global emergence of organisms possessing mutations for resistance to carbapenem antibiotics presents a major threat to public health. Through mechanisms such as the production of *β*-lactamase enzymes, production of efflux pumps, and alterations in porins and penicillin-binding proteins, bacterial species can develop resistance to commonly used antimicrobial agents [1]. These resistance mechanisms can subsequently be transferred to other species by processes including horizontal gene transfer via plasmids, allowing the potential for strains containing multiple resistance mechanisms [2]. The existence of multidrug-resistant organisms with multiple resistance mechanisms presents challenges to proper antimicrobial therapy with important implications in patient care.

One of the most prevalent classes of antimicrobial resistance is the extended-spectrum beta-lactamase (ESBL) class of beta-lactamases, which are capable of hydrolyzing extended-spectrum cephalosporins. Among these, the cefotaximase (CTX-M) class of ESBL enzyme has some of the greatest variance and contributes to the global dissemination of ESBL species. Traditionally, carbapenems have been the preferred treatment of ESBL-producing *Enterobacterales* [3, 4]. These species become increasingly challenging to treat when they acquire carbapenemases as such enzymes hydrolyze the carbapenem antibiotics commonly used against these organisms. The New Delhi metallo-*β*-lactamase (NDM) is one such carbapenemase which has the capability of neutralizing nearly all clinically available *β*-lactam antibiotics [2]. The ability of these enzymes to rapidly develop new variants as a result of one-to-two residue polymorphisms has contributed to the great variance in the phenotype of this carbapenemase class.

Aztreonam (AT) has been known to withstand hydrolysis by NDM enzymes; however, this is often inadequate therapy for these species due to the possibility of concomitant production of serine enzymes such as *Klebsiella pneumoniae* carbapenemase (KPC), oxacillinase (OXA)-type *β*-lactamases, and ESBLs [5]. The OXA-48-like carbapenemases are another group of carbapenemases that have spread beyond the Asian continent, with reports published in 2013 describing isolates in the United States detected as early as 2009 [6, 7]. One of the challenges that clinicals are faced with in the treatment of NDM- and OXA-48-like-harboring *Enterobacterales* is the lack of safe and effective therapy as such isolates are resistant in vitro to nearly all *β*-lactam agents which represent the treatment of choice for many infectious syndromes.

Refinement and expansion of antimicrobial therapy is imperative in the face of the threat posed by carbapenem-resistant organisms. Previously, due to the lack of safe and effective therapies, clinicians were forced to use agents such as polymyxins or aminoglycosides which are limited by suboptimal pharmacokinetics, increased toxicity, and lower efficacy [5, 6]. The *β*-lactamase inhibitor avibactam has demonstrated inhibition of *β*-lactam hydrolysis by serine-based *β*-lactamases (e.g., OXA-48-like and ESBLs) but not metallo-*β*-lactamases [8]. Therefore, the combination of avibactam and AT has been used successfully to cover a wide variety of cabapenemase-producing *Enterobacterales* including NDM-harboring isolates as avibactam protects AT from hydrolysis by coexpressed ESBLs while AT is poorly hydrolyzed by NDM and other metallo-*β*-lactamases providing a much needed treatment option [5]. The extended-spectrum cephalosporin cefiderocol is FDA approved for the treatment of complicated urinary tract infections and hospital/ventilator-associated pneumonia and may represent another treatment option. Cefiderocol retains activity against carbapenem-resistant infections and has shown resistance to hydrolysis by such enzymes as imipenemase (IMP), NDM-1, and OXA-48-like carbapenemases [1, 5]. Herein, we present a case of *Klebsiella pneumoniae* found to harbor NDM, OXA-48-like, and CTX-M resistance genes in a Midwestern Northern American hospital isolated from a patient with recent hospitalization in the Indian state of Kerala.

## 2. Case Presentation

**Figure 1:**
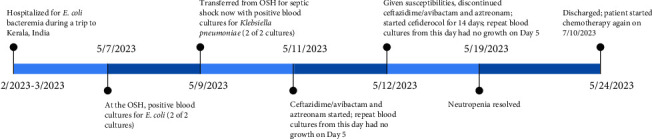
Timeline of blood cultures and antimicrobial treatment.

A 79-year-old female with recent transformation of myelodysplastic syndrome to acute myeloid leukemia and recent initiation of chemotherapy presented to the hospital in the spring of 2023 as an outside hospital transfer. She endorsed an acute onset of abdominal pain and diarrhea and was admitted with neutropenic fever. She developed septic shock and was placed on vasopressors. Her medical history was notable for Type 2 diabetes mellitus, chronic obstructive pulmonary disease, and granulosa ovarian tumor. She had an implanted port-a-cath via right internal jugular vein. She was born in the Indian state of Kerala, where she lived for 30 years prior to emigrating to the United States. She subsequently visited the city of Kumarakom, Kerala, India, frequently, with at least 20 visits lasting from 1 to 4 months in duration. Three months prior to presentation, she visited India and was hospitalized with *Escherichia coli* bacteremia at that time and received antimicrobial treatment (she could not tell the names of the antimicrobial agents). Since her most recent travel, she had one negative set of blood cultures.  Figure 1 describes the timeline of blood culture results and antimicrobial treatment.

Upon initial presentation, she was febrile with a temperature of 39.3°C (102.8°F), tachycardic at 110 beats per minute, hypotensive at 95/56 mmHg, and tachypneic at 25 breaths per minute with desaturation to 85% pulse oximetry. Physical examination revealed mental status change and diffuse lower abdominal tenderness. She was eventually intubated due to worsening mental and respiratory status. Initial laboratory examination revealed leukopenia of 0.1 × 1000 cells/μL, absolute neutrophil count (ANC) 10 cells/μL, thrombocytopenia of 3 × 1000 cells/μL, and anemia with a hemoglobin of 6.7 g/dL. Urinalysis was negative for nitrites, leukocytes, and bacteria. Two sets of blood cultures were collected, and she was initiated on intravenous (IV) vancomycin, cefepime, and gentamicin. She had a computed tomography scan of the chest, abdomen, and pelvis with IV contrast, which was overall unremarkable. The small bowels and colon were normal in caliber and wall thickness, findings not suggestive of neutropenic enterocolitis and colitis. Two of two blood culture sets grew *Escherichia coli* resistant to amikacin, tobramycin, cefazolin, ciprofloxacin and trimethoprim–sulfamethoxazole and intermediate to ampicillin–sulbactam. The blood cultures repeated the next day were negative. An echocardiogram was performed, no vegetation seen. Although her implanted port-a-cath did not appear infected on exam and there was no evidence of thrombophlebitis of right internal jugular vein on venous duplex ultrasound study, it was removed after *Escherichia coli* bacteremia was identified. Further workup for the infections with human immunodeficiency virus, hepatitis C virus, hepatitis B virus, cytomegalovirus, Epstein–Barr virus, and respiratory virus was all negative.

Two days following the original positive blood cultures, two additional sets of blood culture were obtained as the patient had remittent fever with recent history of Indian travel. Both sets of blood culture were positive for Gram-negative bacilli. Multiplex molecular testing was conducted, which detected *Klebsiella pneumoniae* group with detection of CTX-M, NDM, and OXA-48-like resistance genes. She was then initiated on a combination therapy with IV ceftazidime/avibactam (CZA) 2.5 g every 8 h as a 2-h extended infusion plus IV AT 2 g every 8 h as a 0.5-h infusion. Susceptibility testing demonstrated resistance to all *β*-lactam agents (Table 1) as expected based on the genotypic findings. Due to her immunocompromised status and recent antibiotic exposure in India, her multidrug-resistant isolate underwent additional susceptibility testing and was found to be susceptible to cefiderocol (MIC of 2 mg/L using broth microdilution). She was treated with IV cefiderocol 1.5 g every 8 h as a 3-h infusion due to its stability against most *β*-lactamases. The source of bacteremia was thought most likely to be gastrointestinal translocation with colonized strains of bacteria from her prior travel. Risk factors included intubation and orogastric tube insertion during intubation as well as concomitant neutropenia.

She recovered well on her antibiotic regimen and completed 14 days of cefiderocol for the treatment of *Escherichia coli* and multidrug-resistant *Klebsiella pneumoniae* bacteremia. Levofloxacin was subsequently started for bacterial prophylaxis as she remained neutropenic. She was also initiated on opportunistic infection prophylaxis with valacyclovir and posaconazole per protocol.

### 2.1. Diagnostic Assessment

Blood cultures were performed using the BACT/ALERT VIRTUO detection system (bioMérieux) utilizing BACT/ALERT FA Plus and FN Plus (bioMérieux) media. Multiplex molecular testing from positive blood culture media was conducted using BIOFIRE Blood Culture Identification 2 (BCID2) Panel (bioMérieux). Positive blood culture media were subcultured to solid media (Remel), and final identification of organisms was determined by Vitek MS (bioMérieux) matrix-assisted laser desorption ionization time-of-fight (MALDI-TOF) mass spectrometry.

Primary susceptibility testing was performed on the Vitek2 system (bioMérieux) with the AST-GN79 panel. CZA, AT (ETEST, bioMérieux), and meropenem–vaborbactam (MTS, Liofilchem) testing was conducted by gradient diffusion strip. Susceptibility testing for imipenem–relebactam and cefiderocol was conducted by ARUP Laboratories using Sensititre panels (ThermoFisher Scientific). AT and CZA gradient diffusion strips were crossed, as previously described [9], to evaluate for synergistic activity.

## 3. Discussion

The *Enterobacterales* isolate identified in this case demonstrated a multifactorial pattern of resistance mechanisms which presented challenges in appropriate treatment. Considering the patient's travel history to India, it was important to continuously monitor blood cultures instead of confining to the results of first blood culture sets. In the setting of neutropenia and recent antibiotic exposure in India, the potential sources of bacteremia is considered from gastrointestinal bacterial translocation with colonized strains of bacteria from her prior travel despite the absence of imaging findings to suggest enterocolitis. Port-a-cath infection must be considered a possible source and it should not limit to thrombophlebitis. It is suspected that the patient became colonized by multidrug-resistant *Klebsiella pneumoniae* while she was hospitalized in India. A 2019 publication describing the prevalence of carbapenemase genes in South India, including NDM-1 and OXA-48, found that of 370 *Klebsiella pneumoniae* isolates characterized, nearly 14% harbored carbapenemase genes. Of these, 7 isolates were identified with both NDM-1 and OXA-48 carbapenemase [10]. In the United States, *Enterobacterales* demonstrating nonsusceptibility to carbapenems is far less common. Prevalence data from 2022 demonstrated that of 34,623 *Enterobacterales* isolates submitted from 86 hospitals in the United States from 2016 to 2020, 1.3% were nonsusceptible to carbapenems, with KPC being the most commonly detected resistance mechanism. Of these 450 carbapenem nonsusceptible isolates, OXA-48-like carbapenemases were present in 7 isolates and metallo-*β*-lactamases such as NDM-1 and NDM-5 were present in only 12 isolates, and no isolates with either NDM-1 or OXA-48-like genes were identified in the west north central region where the present case occurred [11]. While the pattern of resistance found in this isolate is not uncommon in South India, it is very unusual for a Midwestern U.S. hospital.

Multidrug-resistant Gram-negative pathogens including NDM- and OXA-48-harboring isolates present a significant therapeutic challenge as cross-resistance to virtually all *β*-lactam antimicrobials, thus leaving few safe and effective treatment options. Recent data suggest the role of the combination of CZA plus AT as effective therapy for NDM-harboring *Enterobacterales* because avibactam protects AT from coexpressed ESBL enzymes that readily hydrolyze monobactams while AT is not efficiently hydrolyzed by NDM or OXA-48. Since a commercialized AT/avibactam product was not currently available, avibactam had to be coadministered with ceftazidime [5]. Previous in vitro studies have described the PK/PD relationship of coadministration of this triple combination, which demonstrated the most pronounced bactericidal activity that persisted over a 7-day in vitro model and administration of 8 g per day of AT was preferred [12]. Indeed, the optimal dosage for each agent in this combination regimen remains unknown; however, coadministration of the highest tolerated clinical doses due to the relatively good safety profile of these agents seems warranted [8, 13, 14]. Similarly, administration of prolonged infusion of both products may optimize PK/PD target attainment as the PK/PD driver of efficacy for AT is free-time above the MIC and the PK/PD driver for avibactam protection *β*-lactamases is free time above a threshold concentration [15]. The combination of AT/avibactam was approved by the European Commission in April 2024 for patients with multidrug resistant infections and limited treatment options, cited to be able to be used for complicated intra-abdominal infection, hospital-acquired pneumonia including ventilator-associated pneumonia, and complicated urinary tract infections. The dose of AT/avibactam differs from that used in this case and is specifically designed to optimize the aforementioned PK/PD drivers for both agents [16]. The absence of ceftazidime administration is unlikely to affect the efficacy as AT/avibactam MICs are similar when tested with and without ceftazidime [17]. Conversely, increasing literature describes the importance of complementary PBP inhibition in antibacterial activity; however, in vitro and in vivo models support the antimicrobial activity of AT/avibactam [12, 15].

**Figure 2:**
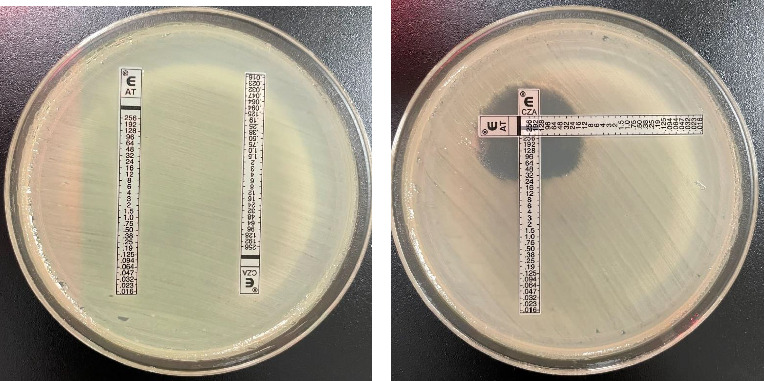
(a) The e-test MIC results for ceftazidime/avibactam (CZA) and aztreonam (AT) alone which shows high-level resistance to each agent. (b) Results from the “Zone of Hope” [9] assessment showing increased clearing in the area where ceftazidime/avibactam and aztreonam strips are crossed. This clearing represents enhanced activity of aztreonam when combined with ceftazidime/avibactam.

Although literature supports the use of the nonconventional combination of CZA plus AT for the treatment of MBL-harboring *Enterobacterales*, one challenge in clinical practice is the lack of a standardized method for assessing in vitro potency of this combination. Previously defined methods such as the “Zone of Hope” use supplies common to clinical microbiology labs to assess enhanced zone of inhibition when gradient diffusion MIC test strips are crossed [9]. For our isolate, Figure 2 shows a clearing between the CZA and AT test strips providing biologic plausibility to the microbiological activity although definitive susceptibility testing results cannot be inferred. Conversely, cefiderocol is a novel antimicrobial agent FDA approved for the treatment of complicated urinary tract infection and hospital/ventilator-associated bacterial pneumonia. Susceptibility testing is challenging as iron-depleted media is required; however, like the present case, susceptibility interpretation can be provided. Large-scale surveillance data show the significant potency of cefiderocol, including among carbapenem-resistant isolates, due to its unique mode of entry into the bacterial cells and stability against many *β*-lactamase enzymes [18]. Interestingly, previous reports have described relatively higher MICs in the presence of NDM enzymes, which was true for our *Klebsiella pneumoniae* isolate that was susceptible to cefiderocol per CLSI interpretation with an MIC of 2 mg/L [19, 20]. The clinical dose of cefiderocol produces sufficient exposure to target MICs up to 4 mg/L [21] which is concordant with the positive clinical outcome described in this case.

## 4. Conclusion

Although the prevalence of NDM- and OXA-48-harboring *Enterobacterales* remains relatively low in the United States, the ease of international travel necessitates a keen awareness of both the risk posed by these organisms and available treatment options. Similarly, clinical implementation of molecular diagnostics may increase the detection of such organisms. Direct from positive blood culture molecular platforms like the BCID used in this case can prompt clinicians to initiate the most likely active antimicrobials (i.e., cefiderocol or CZA/AT) when such organisms are detected to reduce time to active therapy. Indeed, implementation of such platforms in combination with active antimicrobial stewardship is needed to ensure these results are acted upon in a timely manner in an effort to improve clinical outcomes [22]. As such technology undergoes broader implementation, assessment of the impact on time to active therapy and clinical outcomes is warranted. Multidisciplinary initiatives are needed to optimize antimicrobial treatment strategies.

## Figures and Tables

**Table 1:** Antimicrobial susceptibility testing results of *Klebsiella pneumoniae* harboring NDM, OXA-48, and CTX-M.

Antimicrobial	MIC, mg/L (susceptibility interpretation)
Ceftriaxone	≥ 64 (R)
Piperacillin/tazobactam	≥ 128/4 (R)
Cefepime	≥ 64 (R)
Meropenem	≥ 16 (R)
Meropenem/vaborbactam	ND (R)
Ceftazidime/avibactam	> 256/4 (R)
Imipenem/relebactam	≥ 32/4 (R)
Aztreonam	> 256 (R)
Cefiderocol	2 (S)
Tobramycin	≥ 16 (R)
Amikacin	≥ 64 (R)
Gentamicin	≥ 16 (R)
Eravacycline	4 (N/a)
Tigecycline	1 (N/a)
Ciprofloxacin	≥ 4 (R)
Sulfamethoxazole/trimethoprim	≤ 20 (S)

Abbreviations: N/a, not applicable; R, resistant; S, susceptible.

## Data Availability

The data used to support the findings of this study are included within the article.
